# Ruptured rudimentary horn pregnancy mimicking abdominal gestation: A case report

**DOI:** 10.1016/j.radcr.2025.12.030

**Published:** 2026-01-15

**Authors:** Temesgen Getachew, Suleiman Ayalew Belay, Michael A. Negussie, Elezer Berhanu Zewde, Misganaw Abere, Solomon Berihe

**Affiliations:** a Department of Gynecology and Obstetrics, School of Medicine, College of Medicine and Health Sciences, University of Gondar, Maraki Street, Gondar City, Central Gondar Zone, P.O. Box 196, Gondar, Ethiopia; b School of Medicine, College of Medicine and Health Sciences, University of Gondar, Maraki Street, Gondar City, Central Gondar Zone, P.O. Box 196, Gondar, Ethiopia; c School of Medicine, College of Health Sciences, Addis Ababa University, Tikur Anbessa Specialized Hospital, Churchill Avenue, Lideta Sub-City, P.O. Box 5657, Addis Ababa, Ethiopia

**Keywords:** Ectopic pregnancy, Hemoperitoneum, Rudimentary uterine horn, Unicornuate uterus, Uterine rupture

## Abstract

A unicornuate uterus with a rudimentary horn, resulting from incomplete development of one of the Müllerian ducts, is a rare anomaly seen in about 0.4% of women and carries a high risk of ectopic pregnancy and uterine rupture, particularly when the horn is noncommunicating. A 27-year-old primigravida at 16 weeks’ gestation presented with symptoms of hypovolemic shock and peritonitis. Abdominopelvic ultrasound demonstrated an empty uterine cavity with a viable extrauterine fetus, leading to a presumed diagnosis of abdominal pregnancy, and emergency laparotomy confirmed a ruptured pregnancy in a noncommunicating rudimentary horn with significant hemoperitoneum. Surgical removal of the horn and left fallopian tube was performed, and the patient recovered well. This case highlights a common diagnostic pitfall, as rudimentary horn pregnancy is frequently misidentified on ultrasound as an abdominal or intrauterine gestation. Recognition of key ultrasonographic features, including lack of continuity with the endometrial cavity, a myometrial mantle surrounding the gestational sac, and a vascular pedicle, may facilitate earlier diagnosis and reduce the risk of catastrophic rupture. Due to the diagnostic challenges posed by this condition, early identification using advanced imaging and prompt surgical management are critical to reducing maternal risk.

## Introduction

A unicornuate uterus with a rudimentary horn is a rare condition that arises from the arrested development of one of the 2 embryonic Müllerian ducts and occurs in 0.4% of women [1]. There are 2 types of unicornuate uteri: those with communicating and noncommunicating rudimentary horns, with the latter being more common, comprising 85% of cases [2]. Pregnancy in a rudimentary horn is an extremely rare and potentially life-threatening type of ectopic pregnancy, with an incidence ranging from 1 in 100,000 to 1 in 140,000 pregnancies [3]. It carries a significant mortality risk, with a 50% chance of uterine rupture, and is often diagnosed during laparotomy after the gestational horn ruptures [2]. Symptoms may include abdominal pain and abnormal bleeding, with traditional imaging techniques like transvaginal ultrasound frequently failing to identify the condition due to their limited sensitivity [4]. Misdiagnosis is common because rudimentary horn pregnancy can mimic abdominal, angular, or interstitial gestations on ultrasound, particularly when the continuity between the gestational sac and endometrial cavity is not clearly visualized. Delayed diagnosis can result in life-threatening situations for patients, underscoring the importance of high clinical suspicion and advanced imaging modalities to facilitate timely management [5]

We report a case of a ruptured rudimentary horn pregnancy in a 27-year-old primigravida who was initially diagnosed as having an abdominal pregnancy via ultrasound at 16 weeks. This case emphasizes the diagnostic challenges posed by RHP and highlights key ultrasonographic features that may help differentiate it from other extrauterine gestations.

## Case presentation

A 27-year-old primigravida at 16 weeks' gestation presented to the gynecologic emergency department with a 2-day history of generalized abdominal pain, nausea, vomiting, lightheadedness, and blurred vision, but no vaginal bleeding. No antenatal care follow-up had been conducted, and there was no significant medical or surgical history. On examination, blood pressure measured 90/60 mmHg, heart rate was 124 beats per minute, respiratory rate was 24 breaths per minute, and temperature was 36.4°C. Abdominal examination revealed diffuse tenderness with signs of fluid collection, while pelvic examination showed a closed cervix with no vaginal bleeding. Pallor was also noted.

**Fig. 1:**
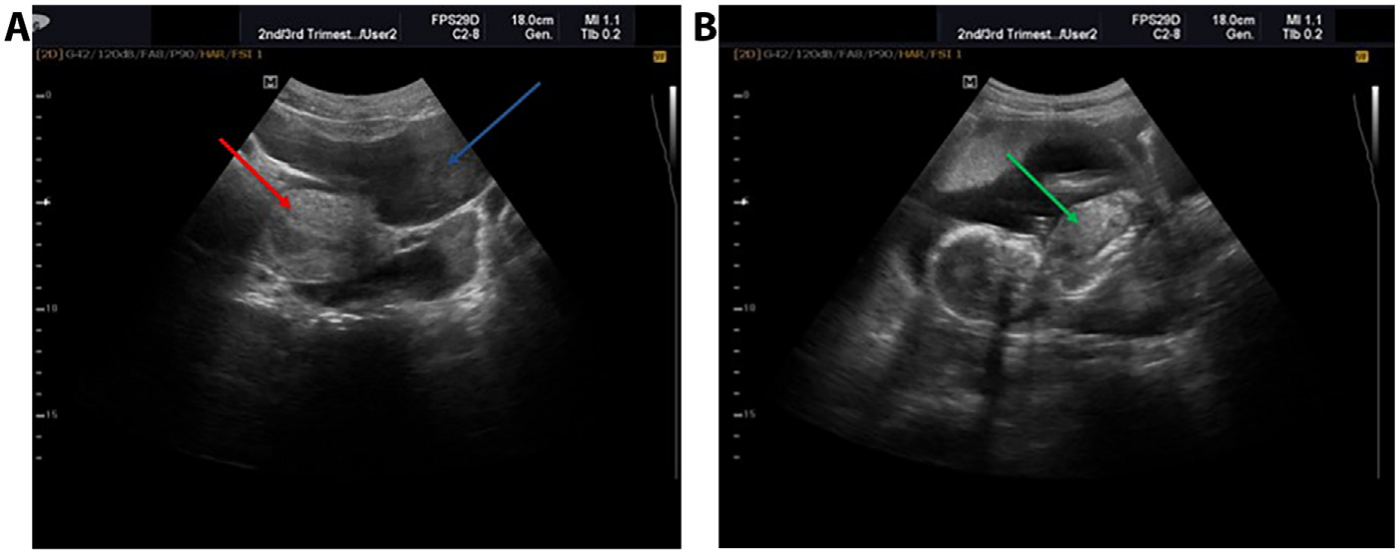
(A) Pelvic ultrasound showing an empty uterus with no intrauterine gestational sac (red arrow) and free peritoneal fluid extending toward Morrison’s pouch (blue arrow), contributing to the mistaken impression of an abdominal pregnancy. (B) Ultrasound image demonstrating a viable extrauterine fetus located outside the uterine cavity (green arrow), leading to an initial diagnosis of abdominal pregnancy before surgical confirmation of a ruptured rudimentary horn pregnancy.

Abdominopelvic ultrasound indicated an empty uterus and a singleton abdominal pregnancy with a positive fetal heartbeat and an estimated gestational age of 18 weeks and 2 days (Fig. 1A and B). The absence of an identifiable myometrial connection to the uterine cavity contributed to the impression of an extrauterine gestation. Free peritoneal fluid extended to the level of Morrison's pouch. Laboratory results showed a hematocrit of 20.1%, while other blood parameters remained within normal limits.

**Fig. 2:**
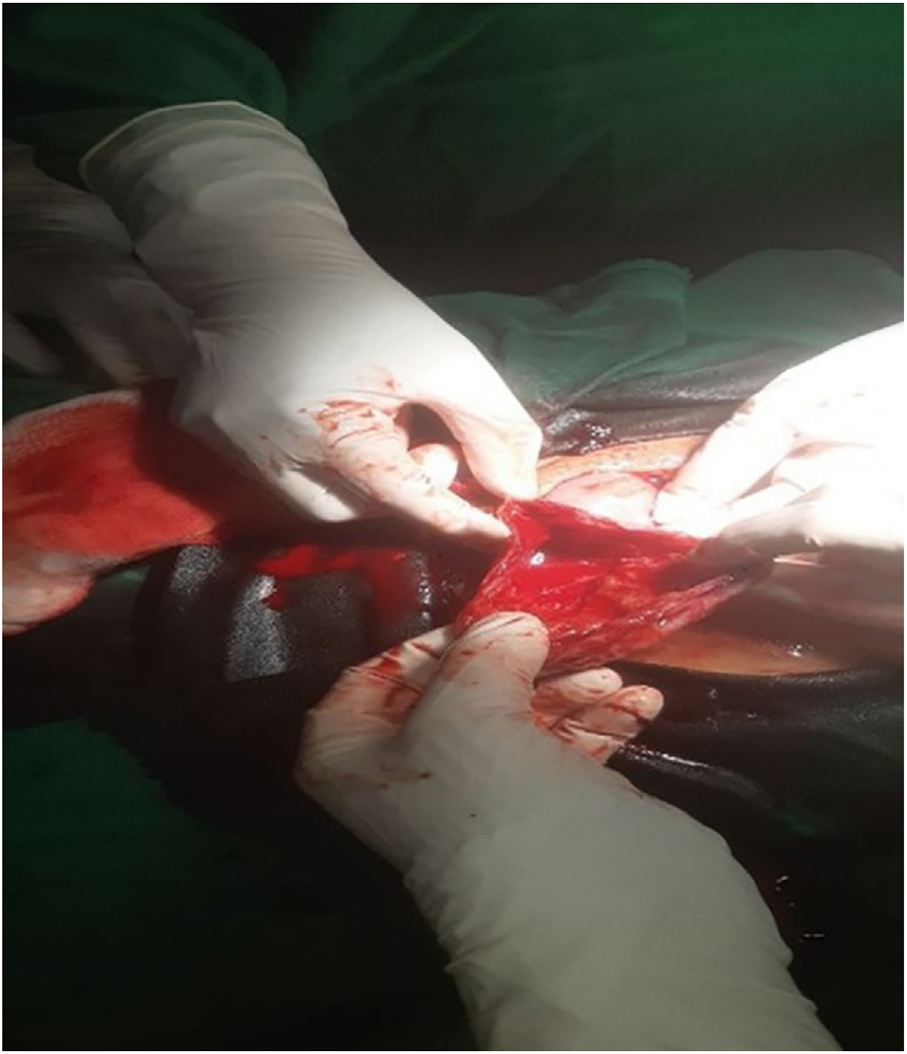
Intraoperative image showing a ruptured left-sided rudimentary horn pregnancy.

With a provisional diagnosis of abdominal pregnancy, an emergency laparotomy was performed. Intraoperatively, the presumed abdominal pregnancy was reassessed and a diagnosis of ruptured rudimentary horn pregnancy was ultimately confirmed. Furthermore, 1.7 liters of hemoperitoneum were discovered, along with a fetus and placenta in the peritoneal cavity. A ruptured left-sided rudimentary horn pregnancy with active bleeding was identified (Fig. 2). There was no connection between the uterus and the rudimentary horn. The fallopian tubes and ovaries appeared normal. Figure 1: (A) Ultrasound image showing an empty uterus. (B) Ultrasound image demonstrating an intra-abdominal pregnancy.

**Fig. 3:**
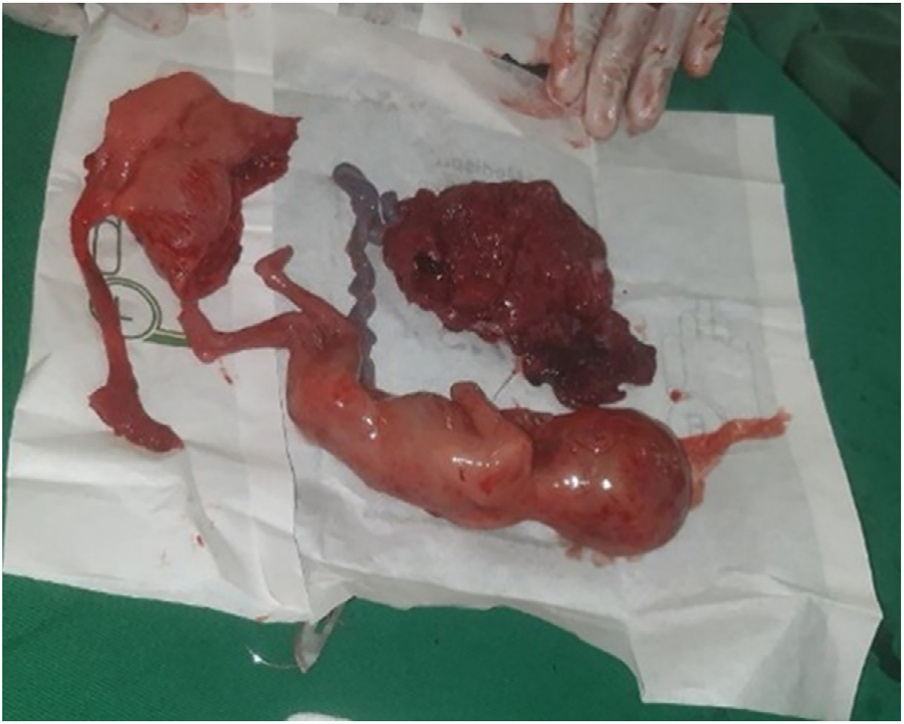
Postoperative image showing resected ruptured rudimentary horn along with removed fetus and placenta.

The hemoperitoneum was evacuated, and the fetus and placenta were removed from the peritoneal cavity (Fig. 3). A cornual resection and left-sided salpingectomy were performed. The patient received 2 units of cross-matched blood, which raised her hematocrit to 27%. She had an uneventful postoperative course and was discharged on the fifth postoperative day with a good outcome. Currently, the patient is following up at our hospital with no complaints.

## Discussion

A unicornuate uterus, which results from partial or complete failure of 1 Müllerian duct to develop, occurs in approximately 1 in 4000 women [1]. About 84% of these cases have a contralateral rudimentary horn [5]. For pregnancy to occur within a noncommunicating rudimentary horn, transperitoneal migration of either a fertilized ovum or sperm from the contralateral tube must occur [2,6,7].

Patients with a unicornuate uterus can have varied clinical presentations ranging from asymptomatic to serious obstetric complications including prematurity, intrauterine growth retardation, recurrent pregnancy loss and higher risks of infertility [1,8]. Furthermore, these patients should be screened for associated urinary anomalies, as an ipsilateral absent kidney is a possible finding. Our patient was asymptomatic, and normal kidneys were confirmed on abdominal ultrasound [6].

Diagnosing this condition is challenging due to the lack of specific clinical criteria and its varied presentation, which can be mistaken for other obstetric conditions [4]. Symptoms may include abdominal pain and abnormal bleeding. This was evident in our patient who presented with a complaint of severe abdominal pain associated with nausea, vomiting, and lightheadedness. Early diagnosis is crucial to prevent complications, as 90% of pregnancies with a rudimentary horn rupture occur between 10 and 20 weeks due to myometrial thinning [2,9,10].

Pregestational diagnosis typically requires hysterosalpingography, hysteroscopy, or laparoscopy, while prenatal diagnosis is often attempted with transvaginal ultrasonography [5,11]. Criteria for diagnosing a rudimentary horn pregnancy include the presence of a myometrium surrounding the gestational sac and absent visual continuity with the uterus and cervix [7]. Additional sonographic clues include a vascular pedicle connecting the gestational sac to the main uterine body and PAS-type peri-sac hypervascularity, findings noted in published descriptions of RHP but not detected in our case. However, the sensitivity of sonography decreases with advancing gestational age, being only 26%. This limitation helps explain why our patient’s 16-week ultrasound incorrectly suggested an abdominal pregnancy. Therefore, an MRI may be needed for confirmation. In our patient, the diagnosis of a ruptured unicornuate uterus was made intraoperatively despite preoperative identification of a 16-week presumed extrauterine (primary abdominal) pregnancy.

Several previously reported cases of rudimentary horn pregnancy highlight diagnostic challenges similar to those faced in our patient [2,5]. Classic reviews and case series consistently note that preoperative diagnosis is often missed, especially in the second trimester, because key sonographic signs, such as a surrounding myometrial mantle, lack of continuity with the endometrial cavity, and the presence of a vascular pedicle, are subtle or obscured as gestation advances [2,5]. Studies also show that ultrasound sensitivity is highest early in pregnancy and decreases significantly after the first trimester, leading to many cases being diagnosed only during rupture at laparotomy [5,7]. Even with Doppler or MRI available, identifying Müllerian anomalies can be more difficult in emergency settings or when hemoperitoneum distorts normal pelvic anatomy [7]. In our case, the 16-week ultrasound suggested an abdominal pregnancy and did not reveal the typical features of a rudimentary horn gestation, ultimately leading to diagnosis during laparotomy. These similarities emphasize that mid-trimester rudimentary horn pregnancies remain inherently difficult to diagnose and require careful assessment of uterine shape, endometrial connection, and adnexal vascular structures during ultrasound evaluations.

The differential diagnosis for a suspected rudimentary horn pregnancy includes several entities that may share overlapping ultrasound features, such as abdominal pregnancy, interstitial (cornual) pregnancy, cesarean scar pregnancy, and angular pregnancy. A structured comparison of key sonographic characteristics distinguishing these conditions is provided in Table 1.

**Table 1:** Differential diagnosis of rudimentary horn pregnancy based on ultrasound features.

Condition	Key ultrasound features	Key clues
Rudimentary horn pregnancy	• No continuity between gestational sac and cervical/main uterine cavity • Myometrium completely surrounds the sac • Vascular pedicle connecting sac to main uterine horn • PAS-type hypervascularity around sac	• Known Müllerian anomaly • Gestational sac located lateral to uterus
Abdominal pregnancy	• Gestational sac outside uterus • Absence of myometrium surrounding sac • Fetus are often mobile or unusually positioned • Placenta implanted on abdominal organs	• Uterus appears empty • Sac not surrounded by uterine tissue
Interstitial (cornual) pregnancy	• Gestational sac in interstitial portion of fallopian tube • Myometrium surrounds sac (>5 mm thickness) • “Interstitial line sign” may be present • Vascular “pedicle sign”	• Eccentric sac high in fundus • Continuity with uterine contour but not with endometrial cavity
Cesarean scar pregnancy	• Sac implanted at site of previous C-section scar • Thin or absent myometrium between sac and bladder • Hypervascularity at scar area • No continuity with endometrial cavity	• History of prior cesarean delivery
Angular pregnancy	• Sac located medial to uterotubal junction • Partial continuity with endometrial cavity • Myometrium often intact around sac	• Sac lies within uterine cavity but eccentrically • Endometrial stripe still seen

Management involves the surgical excision of the pregnant rudimentary horn. Laparoscopy is safe for early, unruptured cases [9,12]. Upon rupture, surgery is necessary for diagnosis and treatment, typically involving excision of the rudimentary horn and ipsilateral salpingectomy to prevent future ectopic pregnancies while preserving the ovary for fertility [12,13]. Recent cases have also combined medical and surgical management, using methotrexate, intracardiac potassium chloride, or lidocaine injections to reduce hypervascularity and complications before surgery [14]. In our case, the patient presented with hypovolemic shock and severe anemia, necessitating laparotomy with cornual resection and salpingectomy. This case highlights the diagnostic pitfall of mistaking ruptured RHP for abdominal pregnancy, reinforcing the need for sonographers and radiologists to actively assess for myometrial continuity, uterine contour abnormalities, and a vascular pedicle when evaluating presumed extrauterine gestations.

Several practical teaching points arise from this case for radiologists. First, when an ultrasound reveals an empty uterus with a live extrauterine gestation, a deliberate search should be conducted for a myometrial rim surrounding the sac, which is one of the key features distinguishing rudimentary horn pregnancy from abdominal pregnancy [7]. Second, the absence of a continuous connection between the gestational sac and the cervical canal should immediately raise suspicion for implantation in a Müllerian anomaly [5]. Third, the presence of a vascular pedicle connecting the gestational sac to the main uterine body on color Doppler is highly suggestive of a rudimentary horn implantation, although this sign may be missed in an emergent setting. Finally, radiologists should recognize that hemoperitoneum and advancing gestational age can obscure these classic features, making early first-trimester imaging and increased clinical suspicion essential to prevent misdiagnosis [2,5,7]. These teaching points can help reduce the likelihood of confusing rudimentary horn pregnancy with abdominal pregnancy, as in our case.

## Conclusion

Ectopic pregnancy in a noncommunicating rudimentary horn is rare and challenging to diagnose. Misdiagnosis is common, especially in the second trimester, when ultrasound sensitivity declines and the presentation may mimic abdominal or other extrauterine gestations. Vigilant care and obstetric expertise are crucial in preventing complications. In this specific case, the patient experienced successful surgical intervention and postoperative recovery, despite a preoperative ultrasound interpretation suggestive of abdominal pregnancy. This case underscores key imaging teaching points, particularly the need to assess for myometrial continuity, a vascular pedicle, and uterine contour abnormalities when evaluating suspected extrauterine gestations. Although the risk for future ectopic pregnancy and uterine rupture persists, necessitating careful monitoring and counseling regarding early imaging in subsequent pregnancies.

## Ethics approval and consent to participate

Not applicable.

## Patient consent

A written Informed consent was obtained from the patient to publish this case report and accompanying images. The editor-in-chief of this journal can review a copy of the written consent upon request.
